# MLKL deficiency in *Braf*^*V600E*^*Pten*^*−/−*^ melanoma model results in a modest delay of nevi development and reduced lymph node dissemination in male mice

**DOI:** 10.1038/s41419-022-04819-4

**Published:** 2022-04-14

**Authors:** Sofie Martens, Nozomi Takahashi, Gillian Blancke, Niels Vandamme, Hanne Verschuere, Tatyana Divert, Marnik Vuylsteke, Geert Berx, Peter Vandenabeele

**Affiliations:** 1 VIB-UGent Center for Inflammation Research, Ghent, 9052 Belgium; 2 Department of Biomedical Molecular Biology (DBMB), Ghent University, Ghent, 9052 Belgium; 3 Cancer Research Institute Ghent (CRIG), Ghent, Belgium; 4 Molecular and Cellular Oncology Laboratory, Department of Biomedical Molecular Biology (DBMB), Ghent University, Ghent, 9052 Belgium; 5 Gnomixx BV, Melle, Belgium

**Keywords:** Experimental models of disease, Cancer models, Melanoma, Necroptosis

## Abstract

Cancers acquire several capabilities to survive the multistep process in carcinogenesis. Resisting cell death is one of them. Silencing of the necroptosis initiator *Ripk3* occurs in a wide variety of cancer types including melanoma. Little is known about the role of the necroptosis executioner MLKL in tumor development. Studies often indicate opposing roles for MLKL as a tumor-suppressing or a tumor-promoting protein. This study investigates the role of MLKL during melanoma initiation and progression using a tamoxifen-inducible melanoma mouse model driven by melanocyte-specific overexpression of mutated *Braf* and simultaneous deletion of *Pten* (*Braf*^*V600E*^*Pten*^*−/−*^). In this model we observed a clear sex difference: melanoma initiation and progression were faster in females mice. *Mlkl* deficiency in male mice resulted in a modest but significant reduction of nevi growth rate compared to the littermate control. In these mice, infiltration and expansion of melanoma cells in the inguinal lymph node were also modestly decreased. This is likely to be a consequence of the delay in nevi development. No significant difference was observed in the *Mlkl*-deficient condition in female mice in which melanoma development was faster. Overall, our results indicate that in this genetic model MLKL has a minor role during melanoma initiation and progression.

## Introduction

Melanoma, a malignant melanocyte neoplasm, is the most aggressive type of skin cancer accounting for 75% of all skin cancer deaths [1]. Even with the emergence of novel immunotherapy, malignant melanoma remains a difficult cancer to treat due to its high plasticity including phenotypic switching [2, 3], heterogeneity [2, 4–6], and therapy resistance. The *BRAF*^*V600E*^ mutation is found in 60% of human melanoma and results in overactivation of the MEK-ERK MAPK pathway [7]. This BRAF^V600E^-induced overactivation of MAPK signaling results in the development of benign melanocytic nevi (hyperplasia) in mice, without progression to malignant melanoma due to oncogene-activated senescence [8]. By additional deletion of *PTEN*, a phenomenon observed in 50% of human metastatic melanoma, the PI3 kinase pathway is activated [7] and malignant transformation is initiated breaking cellular senescence [8]. Melanoma is often resistant to BRAF or MEK inhibitors (vemurafenib or trametinib, respectively) used in mono- or combination treatments [1]. Although the use of immune checkpoint inhibitors is a major improvement in the treatment of advanced melanoma, about 50% of the patients have primary or acquired resistance to them [9]. This immuno-resistance results in further progression of the disease and relapse. To date, conventional cytotoxic treatments do not belong anymore to the standard treatment options of malignant melanoma [10]. Combination of immunotherapy with targeted therapy is a new path sought to overcome therapeutic resistance [9].

Evasion of cell death is one of the major hallmarks of cancer [11]. Downregulation of the gene expression of cell death regulators such as *CASP8* and *RIPK3* are well-known mechanisms that are exploited by cancer cells to evade cell death by apoptosis [11–15] or by necroptosis [16–21], respectively. RIPK3 expression is lost in malignant melanoma and contribute to primary and therapeutic resistance of these cells to necroptosis [22]. Another apoptosis and necroptosis initiator RIPK1 is reported to be the major target of a negative regulation by TAK1, and is directly associated with melanoma progression and metastasis [23]. Little is known about MLKL, the executioner activated by RIPK3 during necroptosis [24], and its opposing roles in cancer. Recent literature suggests that multiple functions of MLKL beyond necroptosis might determine the therapeutic outcome pending on cancer types and stages (reviewed in [25]). Patients with low MLKL levels in pancreatic adenocarcinoma [26, 27], colon cancer [28], gastric cancer [29], cervical cancer [30, 31], and ovarian cancer [32] apparently have worsened overall survival. On the other hand, elevated phospho-MLKL levels have been detected in esophagus and colon cancer [33], suggesting a tumor-promoting role. Other reports also describe higher RIPK3 and MLKL levels in human pancreatic cancer tissue, with MLKL expression even more intense at the tumor invasion front [34, 35]. In low-grade glioma and glioblastoma elevated MLKL expression is correlated with worsened overall survival and disease-free survival [36]. Crucial role of MLKL and necroptosis in mouse inflammation-driven skin cancer was revealed in the rescue of mouse verrucous carcinomas that developed as a result of keratinocyte specific-deletion of Otulin, a signaling molecule that controls TNFR1-mediated cell death [37]. On the other hand, MLKL may play a role in cancer through functions beyond necroptosis [25]. A recent report identified a non-necroptotic function of MLKL in acute myeloid leukemia (AML) that facilitates the release of G-CSF through MLKL-dependent plasma membrane permeabilization without cell death and hereby promoting cancer cell final differentiation. Here, a reduced level of MLKL correlates to poor survival of patients with acute myeloid leukemia (AML) [38]. We studied *Mlkl* deficiency in melanoma initiation and progression in order to better understand MLKL regulation in cancer and its possible function during tumor development. We used the 4-hydroxytamoxifen (4-OHT)-inducible genetic melanoma model in mice (*Tyr::CreERT2;Braf*^*V600E*^*;Pten*^*−/−*^), as this model mimics the genetic profile in human melanoma [8].

## Results

### Sex differences in melanoma development in mice with *Mlkl*^*+/+*^*Braf*^*V600E*^*Pten*^*−/−*^ background

**Fig. 1:**
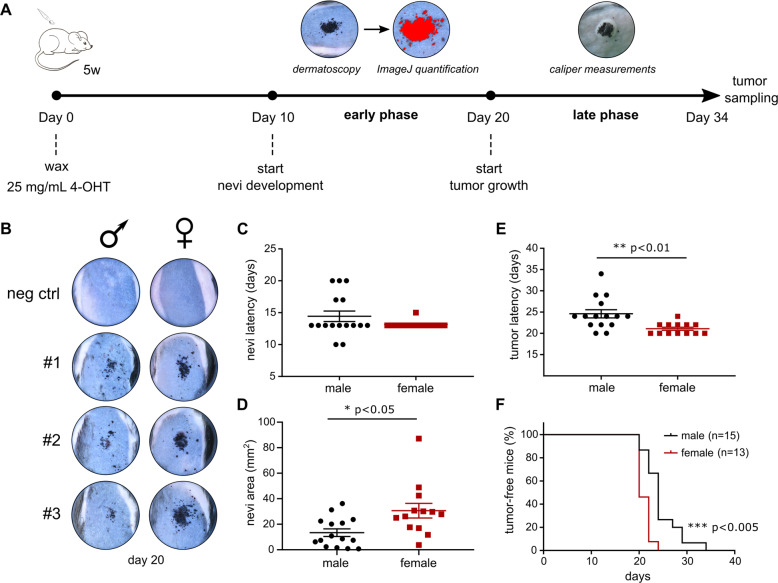
Female mice develop faster melanoma than male mice in the *Mlkl*^*+/+*^*Braf*^*V600E*^*Pten*^*−/−*^ melanoma mouse model. **A** All age-matched mice (5 weeks) (*Tyr::CreERT2*^*+/+*^ *or* ^*tg/+*^*;Mlkl*^*+/+*^ *or* ^*−/−*^*;Braf*^*V600E tg/+*^*;PTEN*^*fl/fl*^*)* were waxed, followed by topical application of 1.5 µL 4-OHT (25 mg/mL) which elicits rapid development of melanoma with 100% penetrance and a short latency of around 3–4 weeks. Tumor development can be divided into two stages: ‘early’ growth of nevi (starting around day 10) which was analyzed by dermatoscopic imaging and ImageJ and ‘late’ vertical tumor growth (starting around day 20) which was analyzed by caliper measurements. Follow-up occurred every other day from day 10 on. Tumor volume was calculated as ‘π/(6*Length*Width*Height)’. **B**–**F** Comparison of melanoma development in female (*n* = 13) and male (*n* = 15) *Mlkl*^+/+^Braf^V600E^Pten^*−/−*^ mice. T-test was performed. **B** Dermatoscopic images of 3 representative nevi per sex group at day 20. Mice with *Tyr::CreERT2*^*+/+*^*;Mlkl*^*+/+*^*;Braf*^*V600E tg/+*^*;Pten*^*fl/fl*^ background, challenged with 4-OHT, are indicated as negative control. Nevi latency (early phase) (**C**) and nevi area (mm^2^) on day 24 (**D**) were compared, the latter calculated using ImageJ. Also tumor latency (late phase) (**E**) and percentage of tumor-free mice (**F**) were compared. *p < 0.05; **p < 0.01; ***p < 0.001.

In order to asses a possible role of MLKL in melanoma initiation and progression, the in vivo conditional *Braf*^*V600E*^*Pten*^*−/−*^ melanoma model of Dankort et al. was used [8]. It is a tamoxifen-inducible mouse model that activates malignant transformation of melanocytes by oncogenic activation of BRAF (BRAF^V600E^ mutation) in the absence of PTEN, a combination that mimics a major process during clinical melanoma progression [8]. Topical application of 4-OHT initiates rapid melanoma development that can be described in two phases: an early phase with initiation and expansion of benign pigmented melanocytic nevi and a late phase with progression to radial and vertical growth of melanoma (Fig. 1A). Mice with *Mlkl*^*+/+*^*;Tyr::CreERT2;Braf*^*V600E*^*;Pten*^*fl/fl*^ background (further referred to as *Mlkl*^*+/+*^ mice) were monitored after 4-OHT treatment over a period of 34 days, as illustrated in Fig. 1A. Sex difference in melanoma development was observed after 4-OHT treatment (Fig. 1B–F) similar to what was recently described in this model [39]. Although there was no statistically significant difference in the onset of nevi appearance (Fig. 1C), female nevi developed faster as is illustrated by a bigger nevi area at day 24 (mean female: 30.62 mm^2^, male: 13.37 mm^2^) (Fig. 1B and D, *p* < 0.05). Onset of the late phase is also earlier in females (Fig. 1E, mean females: 21.08 days, male: 24.6 days, *p* < 0.01), resulting in a significantly reduced tumor-free survival in females (Fig. 1F, *p* < 0.005). Therefore, all further analyses were performed by stratifying the results obtained from males and females.

### MLKL deficiency results in minor delay of nevi development in male mice

**Fig. 2:**
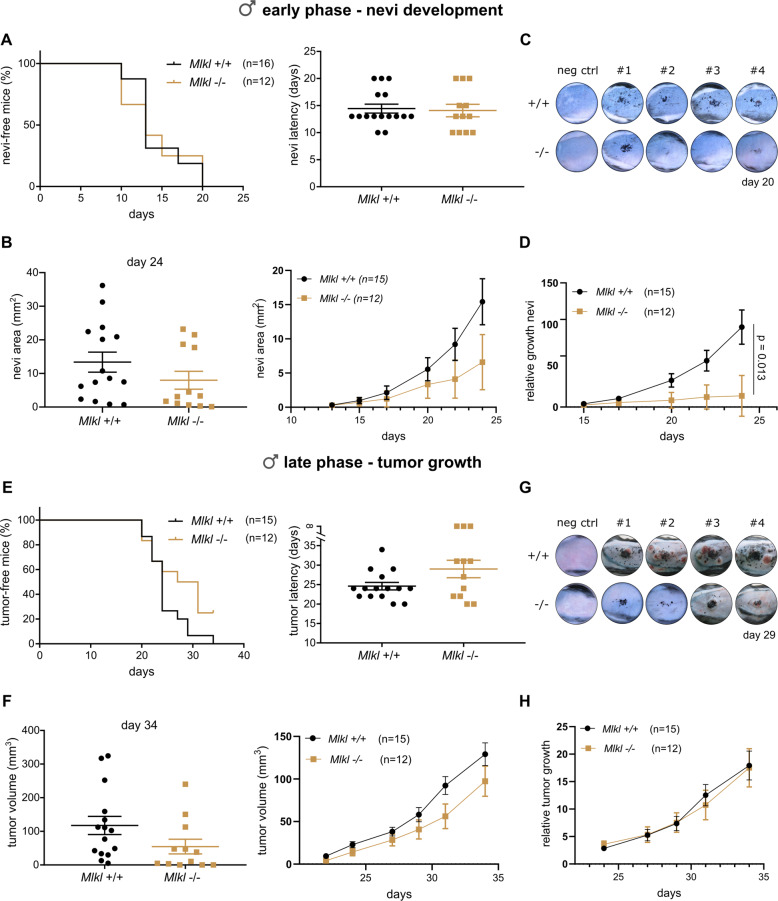
MLKL modestly contributes to nevi growth in melanoma with *Braf*^*V600E*^*Pten*^*−/−*^ background in male mice. Nevi (**A**) or tumor (**E**) latency and percentage of nevi/tumor-free male mice of *Mlkl*^+/+^ and *Mlkl*^*−/−*^ *Braf*^*V600E*^*Pten*^*−/−*^ background were compared. Mean value and SEM are indicated. Log-rank (mantel-cox) test and unpaired two-tailed *t*-test were performed, respectively, but no significant result was obtained. Also, nevi area (mm^2^) (**B**) or tumor volume (mm^3^) (**F**) and relative nevi (**D**) or tumor (**H**) growth were calculated using ImageJ and caliper measurements, respectively. Relative nevi/tumor growth was calculated for each nevi/tumor by dividing its area/volume at every timepoint by the area/volume of the first measurement. The line connects the mean values over time. Synchronization and repeated measurement analysis were performed to analyze differences over time. Nevi area (day 24) (**B**) and tumor volume (day 34) (**F**) are also illustrated (mean value ± SEM). Unpaired two-tailed *t*-test was performed. *p < 0.013. Dermatoscopic images of 4 representative nevi/tumor masses per genotype group at day 20 (**C**) and day 29 (**G**). Mice with *Mlkl*^*+/+*^ or ^*−/−*^ *Tyr*^*:*^*:CreERT2*^*+/+*^*;Braf*^*V600Etg/+*^*;Pten*^*fl/fl*^ background^,^ challenged with 4-OHT, are indicated as negative control.

*Mlkl*^*+/+*^ and littermate *Mlkl*^*−/−*^ mice with a *Tyr::CreERT2*^*tg/+*^;*Braf*^*V600E/+*^*;Pten*^*fl/fl*^ background (further referred to as *Mlkl*^*+/+*^ and *Mlkl*^*−/−*^ mice) were monitored after 4-OHT treatment. Genotyping, qRT-PCR and western blotting confirmed the genetic background of the mice and showed no alteration of *Mlkl* gene expression during melanogenesis and melanoma progression (Supplementary Fig. 2). As mentioned earlier, results were stratified by sex, as a significant sex difference was observed. Although initiation time of the nevi development was similar (Fig. 2A), nevi area tended to be smaller in male *Mlkl*^*−/−*^ mice compared to *Mlkl*^*+/+*^ littermate, albeit not significantly different (Fig. 2B, C). After 24 days *Mlkl*^*−/−*^ nevi reached a mean area of 8 mm^2^ compared to 13.37 mm^2^ in *Mlkl*^*+/+*^ nevi (Fig. 2B). The negative control groups *Tyr::CreERT2*^*+/+*^*;Braf*^*V600E/+*^*;Pten*^*fl/fl*^ did not develop nevi after 4-OHT treatment (Fig. 2C). As neither nevi nor tumor latency was significantly different between the genotypes, experimental data were synchronized to the same start day, being day 13 and day 20, respectively, for early and late phase to allow repeated measurement analysis. This analysis confirmed the sex difference also in terms of nevi growth (Supplementary Fig. 2). In male mice, *Mlkl* deficiency significantly decreased relative nevi growth in the *Braf*^*V600E/+*^*Pten*^*−/−*^ background (*p* = 0.013, Fig. 2D and Supplementary Fig. 2). The relative growth was calculated by normalizing the area of a nevi at each timepoint with the first measurement. In males *Mlkl*^*+/+*^ mice developed pigmented lesions (vertical tumor growth) with a latency of 24 days, while this was 29 days for *Mlkl*^*−/−*^ mice (Fig. 2E). Three *Mlkl*^*−/−*^ mice did not reach the vertical tumor growth phase by the end of the experiment (Fig. 2E) likely reflecting a modest delay in nevi development. Latency of tumor growth was not significantly different between the genotypes due to high variability (Fig. 2E). Tumor volume and relative tumor growth were determined over a time course of 15 days, starting from day 20. *Mlkl* deficiency did not alter melanoma growth significantly in this late phase (Fig. 2F–H). No statistically significant difference was detected in female mice in any of the parameters analyzed (Supplementary Figs. 2–4). Our results illustrate that absence of MLKL has a minor but significant impact on the earlier phase of nevi development in male mice in this model. It is not clear whether the role of MLKL is sex-dependent or dependent on the speed of melanoma progression.

### Cell death is not involved in *Braf*^*V600E*^*Pten*^*−/−*^ melanoma progression

**Fig. 3:**
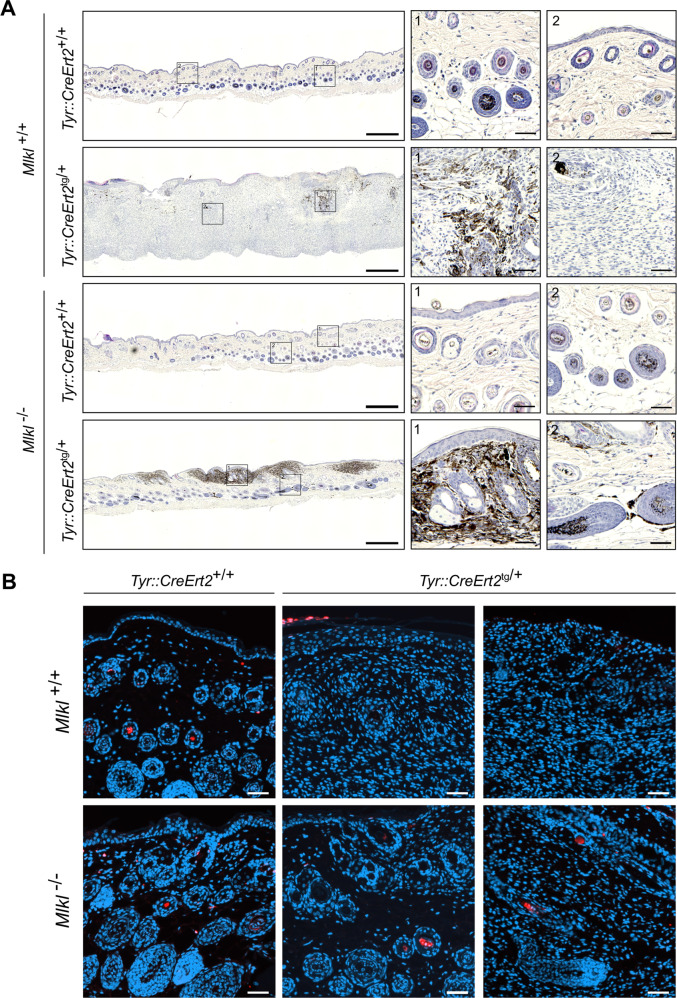
MLKL deficiency in Braf^V600E^Pten^*−/−*^ male mice results in a delay in nevi development, but does not alter tumor mass architecture and intra-tumoral cell death events. Hematoxylin and eosin staining (H/E) (**A**) and TUNEL staining (cell death: red, nuclei: blue) (**B**) of tumor tissue collected at day 36 after 4-OHT treatment. One (H/E) or two (TUNEL) representative tumor sections are shown for each genotype with *Tyr::CreERT2*^*+/+*^ *or* ^*tg/+*^;*Braf*^*V600Etg/+*^*;Pten*^*fl/fl*^ background. Images were taken with a Slide Scanner Axio Scan (Zeiss) and analyzed using ZEN (blue) software (Zeiss). Scale bar in the overview picture represents 500 µM, scale bar in the inserts represents 50 µM. **B** Scale bar represents 50 µM.

RIPK1, RIPK3, and MLKL might have different necroptosis-(in)-dependent roles during melanoma development. RIPK1 is described as an oncogenic driver in melanoma due to its scaffold function independent of the kinase function, while RIPK3 expression apparently is suppressed by the BRAF and AXL oncogenes [40–42]. As there was a minor delay in nevi development in our male *Mlkl*^*−/−*^ mice, we explored further a possible link between necroptosis and melanoma growth in this model. Histological images (H&E staining) illustrate similar tumor mass architecture (late phase) between *Mlkl*^*+/+*^ and *Mlkl*^*−/−*^ male mice (Fig. 3A, Supplementary Fig. 5). *Mlkl*^*+/+*^ male mice developed melanocytic hyperplasia after 4-OHT treatment with a pagetoid spread of pigmented melanoma cells into the epidermis and spread throughout the dermis (Fig. 3A). Though tumor architecture was similar in *Mlkl*^*+/+*^ and *Mlkl*^*−/−*^ mice, about 50% of male *Mlkl*^*−/−*^ *mice* was in an earlier stage of melanoma development with smaller tumor mass (Fig. 3A). Histological analysis detected no necrotic areas in these melanoma lesions. TUNEL staining indicated little, if any, cell death events in the *Mlkl*^*+/+*^ and *Mlkl*^*−/−*^ tumor (Fig. 3B). Overall, this shows that cell death is a rare event in melanoma lesions of this model and there was no difference in *Mlkl*-deficient condition. This may suggest no cell death-dependent functions of MLKL involved in nevi development in this genetic model. Finally, also *Ripk3*^*+/+*^ and *Ripk3*^*−/−*^ mice with a *Tyr::CreERT2*^*tg/+*^*;Braf*^*V600E/+*^*Pten*^*fl/fl*^ background were included in the study to address further a possible role for necroptosis. Kinetics of Initiation and progression of melanoma was similar in the *Ripk3* background as the *Mlkl* background. RIPK3 deficiency did not alter nevi expansion or tumor growth in either sex (Supplementary Fig. 6) (Supplementary Fig. 7). Therefore, no further analysis on these mice was performed. Unlike MLKL the necroptosis-regulator RIPK3 does not seem to have any impact on melanoma progression in this model. Therefore the minor impact of *Mlkl* deficiency on the nevi growth observed in male mice is likely to be related to a necroptosis-independent function of MLKL.

### *Mlkl* deficiency results in decreased colonization of melanocytic cells in lymph node of male *Braf*^*V600E*^*Pten*^*−/−*^ mice

**Fig. 4:**
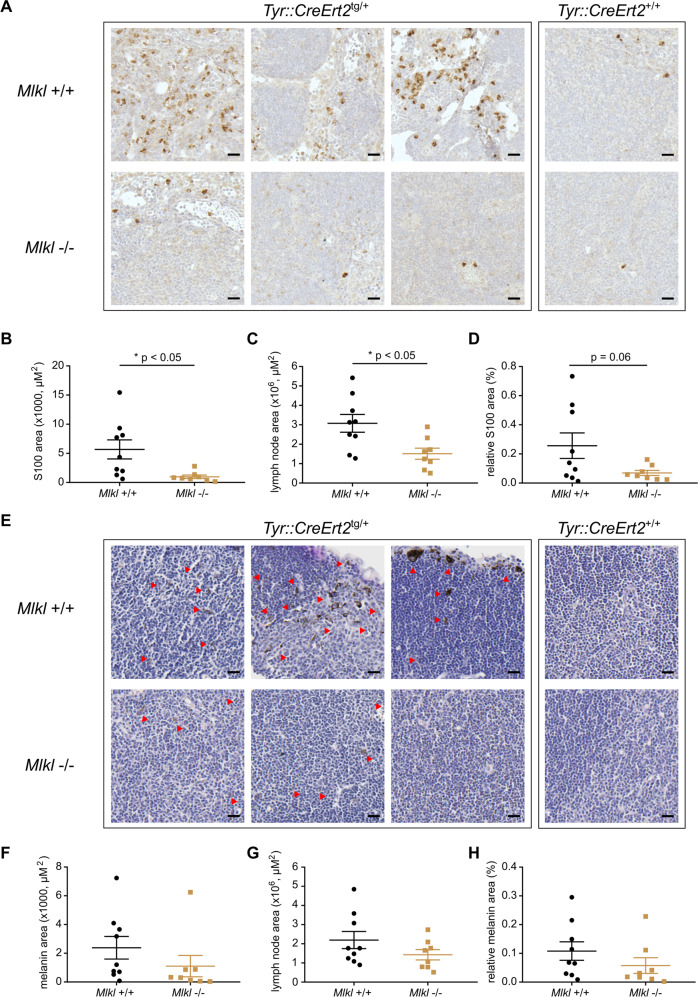
MLKL deficiency in *Braf*^*V600E*^*Pten*^*−/−*^ melanoma delays colonization of melanoma cells in the lymph node of male mice. Histology and quantification of S100 staining (**A**–**D**) and melanin (**E**–**H**) in lymph node tissue collected at day 36 after 4-OHT treatment. Three representative lymph node sections are shown for each genotype with *Tyr::CreERT2*^*+/+*^ *or* ^*tg/+*^*;Braf*^*V600Etg/+*^*;Pten*^*fl/fl*^ background. Images were taken with a Slide Scanner Axio Scan (Zeiss) and analyzed using ZEN (blue) software (Zeiss). Scale bar represents 20 µM. After removal of outliers (removal if value is higher/lower than 2 x SEM), unpaired two-tailed *t*-test was performed for S100/melanin area (**B**, **F**), lymph node area (**C**, **G**), and relative S100/melanin area (S100/melanin area divided by lymph node area) (**D**, **H**). Red arrows indicate melanin-pigmented cells. *p < 0.05.

In order to investigate the effect of *Mlkl* deficiency on melanoma cell dissemination, inguinal lymph nodes were collected after 36 days and lymphatic infiltration was quantified. The melanoma model in C57BL/6 mice is a very fast model and humane endpoint only allows detection of early spread of melanoma cells in the inguinal lymph nodes closest to the 4-OHT-treated skin area. Macroscopic evaluation of the inguinal lymph nodes of male mice showed less melanoma lesion in *Mlkl*^*−/−*^ condition (Supplementary Fig. 8). Indeed, further evaluation of two parameters, presence of the melanin pigment (differentiated melanoma cells) and of S100 protein (all cells with melanocytic origin), confirmed decreased presence of melanoma cells in male *Mlkl*^*−/−*^ lymph nodes (Fig. 4, Supplementary Fig. 9, Supplementary Fig. 10). Not only the size of male *Mlkl*^*−/−*^ lymph node was significantly smaller (*Mlkl*^*+/+*^: 3.08 × 10^6^ µm^2^, *Mlkl*^*−/−*^: 1.5 × 10^6^ µm^2^, *p* < 0.05), but also the total S100 area was smaller (*Mlkl*^*+/+*^: 5.7 × 10^3^ µm^2^, *Mlkl*^*−/−*^: 0.98 × 10^3^ µm^2^, *p* < 0.05) (Fig. 4A–C). Although not statistically significant, similar trends were observed for the relative S100 area (Fig. 4D) and melanin area (Fig. 4E–H). These results might reflect the minor delay in nevi development observed in the early phase. That females have a faster disease progression in our *Braf*^*V600E*^*Pten*^*−/−*^ melanoma model was again confirmed, as larger islands of melanoma cells could be detected in their lymph nodes at the time of dissection (Supplementary Fig. 11 compared to Fig. 4). In female mice no statistically significant difference was observed in *Mlkl*^*−/−*^ condition (Supplementary Figs. 11–13), consistent with the absence of any effect on nevi growth. Overall, we conclude that a modest reduction in melanoma lymph node infiltration observed in *Mlkl*^*−/−*^ male mice is likely to be a consequence of the delayed nevi growth.

## Discussion

To date, the functional role of the necroptosis executor MLKL in tumor development has not been intensively studied (reviewed in [25]). It is not clear whether necroptosis in tumor cells can contribute to a pro- or anti-tumorigenic microenvironment [43]. In order to better understand pathophysiological functions of MLKL in tumor development, we studied the impact of *Mlkl* deficiency on melanoma development using the *Braf*^*V600E*^*Pten*^*−/−*^ genetic mouse model. Expression of necroptotic molecules may vary depending on the cancer type and stage (reviewed in [25]). For example, RIPK3 expression is mild in early stages of MMVT-PyMT mammary gland cancer, but increases in the late stages of the disease [44]. In our model, MLKL was still detectable in the primary tumor, which suggests no major downregulation during melanoma development in this model. Not only expression levels, but also mutations could disable necroptosis in melanoma. For example, a *RIPK3* V458M mutant was found in melanoma (COSMIC) that is potentially defective in its RHIM domain [43]. Many somatic mutations in *MLKL* are also found in malignant melanoma patients, of which many are located in the pseudokinase domain (D390N, F469Y, P189L, S454F, L377F, G330E, D296N, P260L, E250K, F398I, L291P, E351K) and a few in the 4-helical bundle domain (G74E, E70K, P41A) (COSMIC) [43, 45, 46]. The L291P mutation was found in human stomach adenocarcinoma [47] and results in a loss-of-function in mouse L929 cells (corresponding L280P in mice) [47]. This suggests a potential role of MLKL-dependent cell death in development of adenocarcinoma. G330E and E351K mutations located in the pseudokinase domain compromise liposome-permeabilization assay as well as necroptosis assay in human U937 cells [48]. The functional consequences of other mutations and their role in disease progression is not known. As our tamoxifen-inducible melanoma model is a very fast model ending around 36 days after initiation, a contribution of additional *Mlkl* mutations in our model are unlikely.

We showed that full *Mlkl* deletion caused a modest but significant delay in relative nevi growth, while the latency of nevi appearance was identical in *Mlkl*^*+/+*^ and *Mlkl*^*−/−*^ mice. In contrast, no significant difference was observed in female mice. It is unclear whether this indicates a male-specific role for *Mlkl* or the effect is masked by faster melanoma development in female mice. The sex difference we observed is consistent with an earlier report [39] and likely to involve sex-dependent signaling in melanoma or use of tamoxifen (estrogen receptor modulator) as the inducer in this model. The impact of *Mlkl* deficiency on the constitution of the tumor microenvironment and therefore its impact on possible anti-tumor immune responses in this fast melanoma model are not known and might need other experimental set-ups to further investigate. For this, we could consider the *Tyr::Nras*^*Q61K*^ melanoma model characterized by a hyperpigmented skin resembling giant congenital nevus and a much longer latency for melanoma formation compared to the *Braf*^*V600E*^*Pten*^−*/*−^ [49]. Full *Ripk3*^*−/−*^ mice did not behave differently from littermate *Ripk3*^*+/+*^ mice in the *Braf*^*V600E*^*Pten*^*−/−*^ melanoma model. Although RIPK3 is lost in a panel of malignant melanoma cells and functions as a suppressor of melanomagenesis [22], it does not seem to play any role in our genetic model. Additionally, neither necrotic core nor cell death was detected in the solid, primary melanoma tumor. These results further indicate that necroptosis most likely does not contribute to tumor initiation and progression in *Braf*^*V600E*^*Pten*^*−/−*^-driven melanoma, in contrast to many other solid tumors that rely on MLKL-dependent cell death to promote tumor growth and metastasis [44]. The slower nevi growth rate in *Mlkl*^*−/−*^ mice is translated into a tendency of delayed onset of the vertical tumor growth phase, although not statistically significant. *Mlkl* deficiency in tumor cells themselves or in the immune cells invading the tumor microenvironment has been described to promote [16, 31, 50–53] or delay tumor growth [33, 35, 54]. For example, MLKL induces necroptosis in immunosuppressive tumor-associated macrophages (TAMs) [31] and *Mlkl* downregulation in acute myeloid leukemia (AML) cells aggravates the disease in mice [50]. The latter case was recently shown to be attributable to MLKL’s function regulating G-CSF release through a plasma membrane pore and was independent from necroptotic function [38]. On the other hand, RIPK1/3- and MLKL-dependent necroptosis in pancreatic ductal adenocarcinoma (PDA) cells would recruit and activate immunosuppressive TAMs and myeloid cells [35, 55]. Despite the possible roles of necroptosis modulators in regulating tumor progression and tumor microenvironment in several cancer types, we could not identify a major tumor-promoting or tumor-suppressive role for MLKL in primary melanoma development beyond the melanocyte hyperplasia only seen in male mice. However, the effect of *Mlkl* deletion on the relative nevi expansion was significant in a robust statistical analysis. It clearly differed from *Ripk3* deficient mice where all parameters measured remained identical compared to its littermate control (Supplementary Fig. 2 vs. Supplementary Fig. 7). In interpreting data with a subtle difference, thorough control of the experimental conditions is crucial. Inter-experimental variability and differences in experimental conditions such as genetic background of mice and use of non-littermate control can lead to different conclusions and controversies in literature [56]. For this reason we unified the background of mouse lines by backcrossing when necessary and matched littermates of each line were used as a control (see the “Materials and methods” section). We also took into account of inter-experimental variabilities in statistical analysis (see “Materials and methods”). The impact of *Mlkl* deficiency on relative nevi growth was also consistently reflected in a tendency of smaller tumor size, reduced tumor incidence, and increased tumor-free survival rate. 3/12 (25%) *Mlkl*^*−/−*^ mice did not develop tumoral lesions during the time of observation. This might point to a possible role of MLKL in regulating cellular senescence and opens a vastly unexplored area. Our data also demonstrate less colonization of melanoma cells in the lymph nodes of *Mlkl*^*−/−*^ mice. This is most likely a consequence of the minor delay in nevi development observed. Currently, we cannot conclude whether MLKL directly contributes to melanoma dissemination in our model, even though numerous reports established its role in metastasis of various solid tumors. For example, MLKL is linked to necroptosis in necrotic areas of solid tumors, an essential process that shapes the tumor microenvironment and promotes metastasis [44]. *Mlkl* deficiency impaired metastasis in an orthotopic breast cancer mouse model [44] and also in a lung colonization model of nasopharyngeal carcinoma [57]. Mechanistically MLKL-induced necroptosis would not contribute to the migration capacity of cells, but rather contributes to an inflammatory microenvironment that favors metastasis in vivo [34, 44]. Of note, MLKL is described to play a role in epithelial-to-mesenchymal transition (EMT) and contribute to the invasive behavior of radioresistant nasopharyngeal carcinoma cells through induction of EMT genes, a function that is suggested to be independent of its phosphorylation status [57]. Finally, MLKL can also activate cell-surface proteases (ADAM family) that in their turn promote invasion of colon cancer cells [58]. These reports, together with our results, indicate that role of MLKL in invasion and metastasis might depend on tumor type and stage, and might involve necroptosis-independent functions.

As no cell death-inducing therapies exist for the treatment of melanoma patients [59, 60], gaining more insights into the pathways controlling necroptosis resistance in invasive melanoma populations will offer opportunities to design novel therapies for melanoma. Therapeutically targeting MLKL’s function as the necroptosis executioner proved to be promising. For example, intra-tumoral delivery of MLKL mRNA in mice delays the growth of primary B16 melanoma tumors due to necroptotic cell death and consequent activation of the immune system to attack tumor neo-antigens [61]. Our results suggest only a minor role of MLKL in melanomagenesis in the current genetic model we used. MLKL might have cell death-independent functions that contribute to early nevi growth in vivo. Whether or not the early lymph node infiltration of melanoma cells simply reflects the delay in nevi growth or suggests a role in dissemination, its possible impact on the final disease progression remains an open question. Seen the vital role of the lymphatic system in the process of melanoma metastasis and the prognostic value of the sentinel lymph node dissemination [62], this relatively marginal observation might imply a higher biological significance and thus warrant further investigation. Unraveling the possible duality of MLKL in melanoma and its cell death resistance mechanisms will contribute to the development of potential therapies beyond necroptosis.

## Materials and methods

### In vivo *Braf*^*V600E*^*Pten*^−/−^ melanoma model

All in vivo experiments were conducted according to institutional, national, and European regulations. Animal protocols were approved by the ethics committee of Ghent University. C57BL/6 *Tyr::CreERT2*^*tg/+*^*;Braf*^*V600E tg/+*^*;Pten*^*fl/fl*^ mice [8] were bred on a *Mlkl*^*+/+*^*, Mlkl*^*−/−*^*, Ripk3*^*+/+*^, or *Ripk3*^*−/−*^ background. C57BL/6J *Mlkl*^*−/−*^mice were a gift from Dr. Warren Alexander and Dr. James Murphy (Walter & Eliza Hall Institute of Medical Research) [63]. *Ripk3*^*−/−*^ mice originally in C57BL/6N background were a gift from Dr. Vishva Dixit (Genentech, San Francisco) [64], but further backcrossed into C57BL/6J background for more than 5 generations in the laboratory, as all other strains were C57BL/6J and we reported earlier marked difference between C57BL/6J and C57BL/6N in various animal models [65]. All mice were maintained by heterozygote breeding to produce littermate controls for the experiments. *Tyr::CreERT2*^*+/+*^*;Braf*^*V600E tg/+*^*;Pten*^*fl/fl*^ mice with identical backgrounds were used as negative controls to demonstrate Cre-specific tumorigenesis. The right sample size was determined by using G*Power software. Due to the limitations of the breeding scheme for this genetic model and use of littermates, mice were stratified instead of randomized to reduce variabilities among cages and groups. In Age-matched mice of 5 weeks old were depilated prior to tamoxifen application in order to synchronize hair follicle cycles. Dorsal hair of anesthetized mice was removed using a (50:50 w/w) mix of beeswax and gum rosin (Sigma-Aldrich, #243221 and #1.0–1.5G). 4-hydroxytamoxifen (4-OHT; 70% Z-isomer, Sigma-Aldrich, #H-6278) was diluted in (1:1 w/w) DMSO:EtOH to a concentration of 65 mM (25 mg/mL) and 1.5 µL 4-OHT (25 mg/mL) was applied with a pipette on the waxed skin area. Mice were dissected at a defined timepoint. If mice suffered from tumor burden or as soon as the tumors reached 1.0–1.5 cm^3^, mice were sacrificed earlier. Treated animals were evaluated weekly for tumor development and progression. Small melanocytic lesions on the skin (early phase characterized by spreading of pigmented melanoma papules) of anesthetized mice were analyzed using dermatoscopy (Canon PowerShot G10, Dermlite dermatoscopic attachment) every other day from day 10 onwards to determine growth rate. Chemical depilation (Veet) was used in order to image the nevi properly. Digital quantification was performed using ImageJ. Tumor volumes (late phase characterized by vertical outgrowth of tumor mass) were measured every other day from day 20 onwards with a caliper and calculated using the formula π/(6*Length*Height*Width). All measurements and evaluations were done by two independent persons without formal blinding. For genotyping, mice tails were incubated ON at 55 °C in 200 µL gDNA lysis buffer (1 M Tris pH 8.8, 0.5 M (NH_4_)_2_SO_4_, 0.05 M MgCl_2_, 0.5% Triton X-100) with 40 µg/mL proteinase K. Next, samples were boiled for 10 minutes at 95 °C and 1 µL DNA sample/PCR reaction was used. PCR was performed using ALLin HS Red Taq Mastermix according to manufacturer’s instructions. The reaction consisted of incubation at 95 °C for 5 min, followed by 30 cycles of 94 °C (1 min), 59 °C (1 min), and 74 °C (1.5 min), finalizing with 74 °C for 10 min. Primers used: MLKL Fw: TATGACCATGGCAACTCACG; MLKL Rev: ACCATCTCCCCAAACTGTGA.

### Histology and TUNEL staining

Formalin-fixed tissue was embedded in paraffin and 4 µM sections were cut and stained with hematoxylin and eosin (H/E). Sections were dewaxed, rehydrated, and incubated with permeabilisation solution (0.1% Tx100 + 0.1% NaCitrate) for 8 min at room temperature. Slides were rinsed with bidi, followed by PBS. Next, cell death was analyzed using in situ cell death detection kit (TMR-red, Roche) according to manufacturer’s instructions. Nuclei were stained with 1 µM Hoechst solution for 30 min at room temperature. Images were taken with the Slide Scanner Axio Scan (Zeiss) and images analyzed using the ZEN (blue) software (Zeiss).

### S100 staining

Formalin-fixed tissue was embedded in paraffin and 4 µM sections were cut, dewaxed, and rehydrated. Sections were depigmented with 10% H_2_O_2_ in 0.05 M PBS (pH 7.4) for 1.5 h at 55 °C before antigen retrieval. Antigen retrieval was performed using citrate buffer pH 6 (Vector Laboratories, H-3300) and PickCell electric pressure cooker. Next, slides were rinsed with PBS (3 × 5 min), followed by blocking of peroxidase for 10 min with 3% H_2_O_2_ in methanol and again rinsed with PBS (3 × 5 min). Blocking was performed with 5% goat serum in 1% BSA in PBS for 30 min. Slides were incubated with primary anti-S100 antibody (1/20000, DAKO, Z0311) in 1% BSA in PBS overnight at 4 °C and secondary goat anti-rabbit Ab-biotin (1/500, DAKO, E0432) in blocking buffer. Rinsing steps were always performed with PBS (3 × 5 min). Finally, slides were incubated with ABC (Vector Laboratories, PK-7100) for 30 min and incubated with DAB until specific staining appeared. Reaction was stopped with tap water and slides were mounted with xylene-based mounting medium. Lymph node area, S100 area, and melanin area (the latter visible on H/E stainings) were quantified using QuPath. Outliers (values higher/lower than 2 x SEM) were removed from the analysis.

### Statistics

Data are means ± standard error of the mean for *n* mice analyzed in two independent experiments, unless indicated otherwise. The log-rank test, unpaired two-tailed *t*-test, and ANOVA were done using GraphPad Prism 7.01. For percentage of nevi-/tumor-free mice, the curve was analyzed with a Log-rank (Mantel-Cox) test. Unpaired two-tailed *t*-test or one-way ANOVA and a Bonferroni multiple comparison test was performed where indicated. Statistical significance was defined as *P* < 0.05. As latency of nevi and tumor development did not differ between *Mlkl*^*+/+*^ and *Mlkl*^*−/−*^, data of nevi area, relative nevi growth, tumor volume, and relative tumor growth were synchronized to reduce variation. Synchronized nevi area and relative growth, and synchronized tumor volume and relative growth were analyzed as repeated measurements using the residual maximum likelihood (REML) approach as implemented in Genstat v21 (VSN International 2021). Briefly, a linear mixed model of the following form (random terms underlined): response = μ + experiment + sex + time + time.experiment + time.sex + time.genotype + time.genotype.sex + time.subject was fitted to the data. The random interaction term time.subject represents the residual error term with dependent errors because the repeated measurements are taken on the same subject, causing correlations among observations. Times of measurement were set at equal intervals, the autoregressive correlation structure (AR), allowing for serial correlation within subjects, was selected as correlation model, and unequal variances across time were allowed. Significances of the fixed main effects, of changes in difference between e.g., sexes and genotype effects over time (two-way interaction terms), and sex-specific differences between genotype effects over time (three-way interaction term) were assessed by an *F*-test. The significance of individual comparisons between the two genotypes within sex was assessed by a *t*-test.

## Supplementary information

Supplemental Material

Checklist

## Data Availability

The datasets used and analyzed during the current study are available from the corresponding author on reasonable request.
