# Supplementary material for: MLKL deficiency in BrafV600EPten−/− melanoma model results in a modest delay of nevi development and reduced lymph node dissemination in male mice
Source: Cell Death Dis. 2022 Apr 14;13(4):347. doi: 10.1038/s41419-022-04819-4 (PMC9010476; doi:10.1038/s41419-022-04819-4)
Supplement: Supplementary file 1 — Supplemental Material [file 41419_2022_4819_MOESM1_ESM.docx]

**Supplementary Information**

**Figure legends and Supplementary Figures**


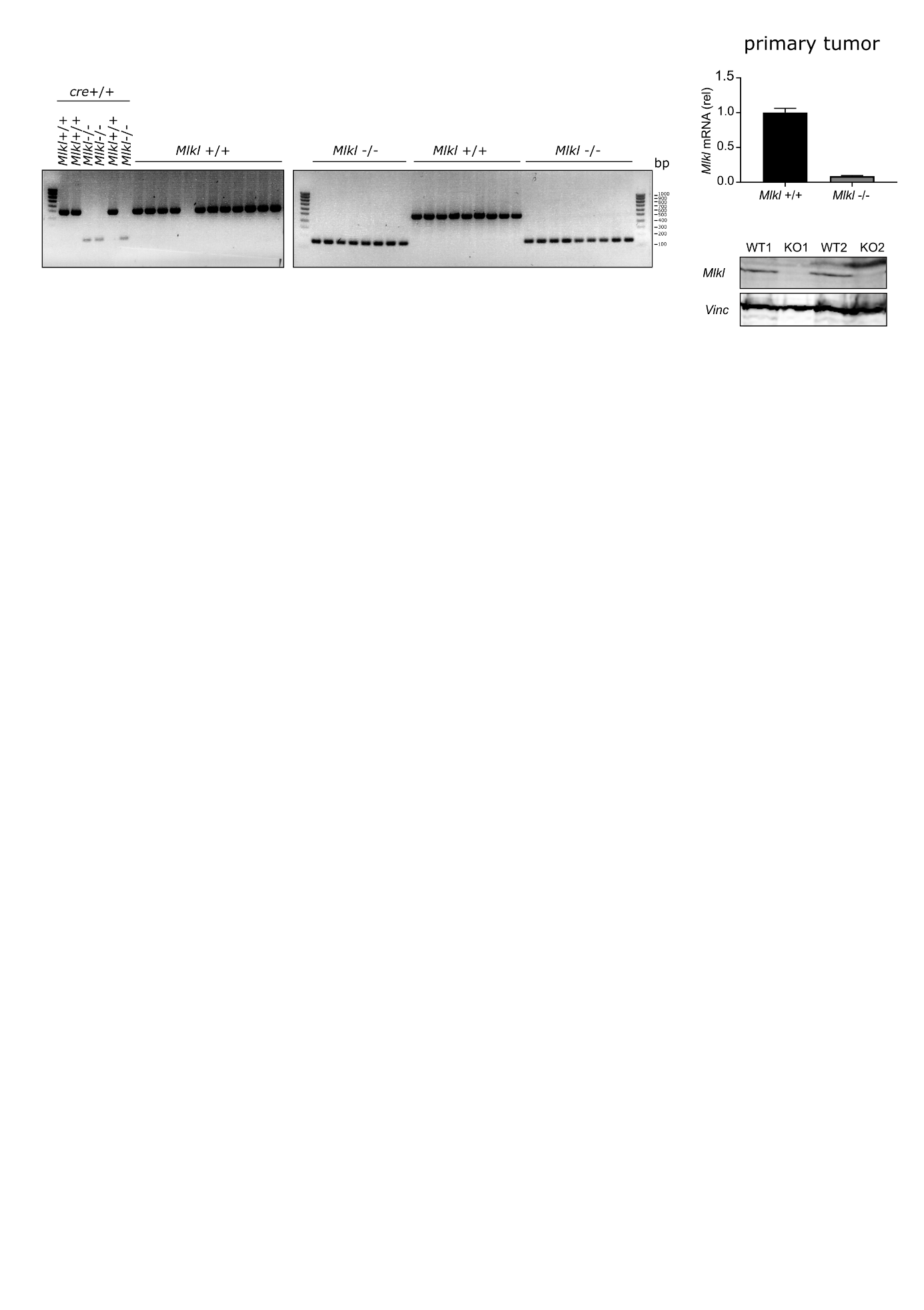


**Supplementary Figure 1. Confirmation *Mlkl* KO in *Braf^V600E^Pten^fl/fl^* and *Tyr::CreERT2^tg/+^;Braf^V600E^Pten^fl/fl^* control mice.** (left) RNA was isolated from the primary tumor at day 36 after 4-OHT treatment and qPCR was performed to confirm *Mlkl* knockout in *Mlkl^-/-^ ;Tyr::CreERT2^tg/+^;Braf^V600E^Pten^fl/fl^* mice. One knock-out mouse compared to one wild type mouse. (right) *Mlkl* genotyping of *Mlkl^-/-^ and ^+/+^ Tyr::CreERT2^tg/+^;Braf^V600E^Pten^fl/fl^* mice. DNA ladder used was Biorad small fragment ladder (100-1000bp). *Mlkl* WT band runs at 499 bp, *Mlkl* KO band runs at 159 bp.

**
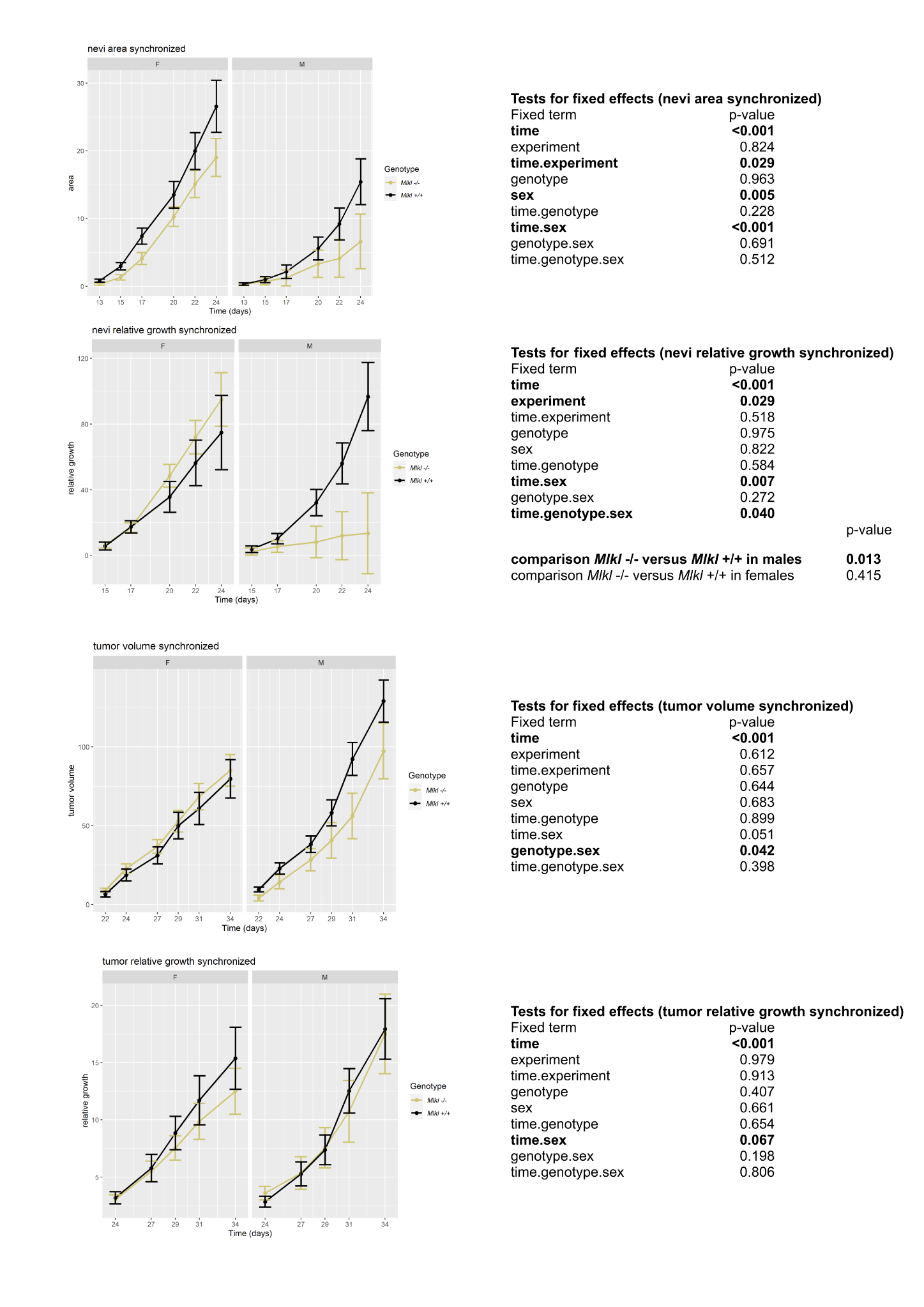
**

**Supplementary Figure 2. Statistical analysis performed on data from Figure 2 and Supplementary Figure 4.** As nevi and tumor latency did not differ, data were synchronized to day 13 or day 23 respectively in order to decrease variation. Repeated measurement analysis was performed by maximum likelihood (REML) approach over time as described in Materials and Methods section. Criteria taken into account in the analysis are: cage variation, experimental variation, sex, time and genotype. P values are indicated. Significant p-values are indicated in bold.


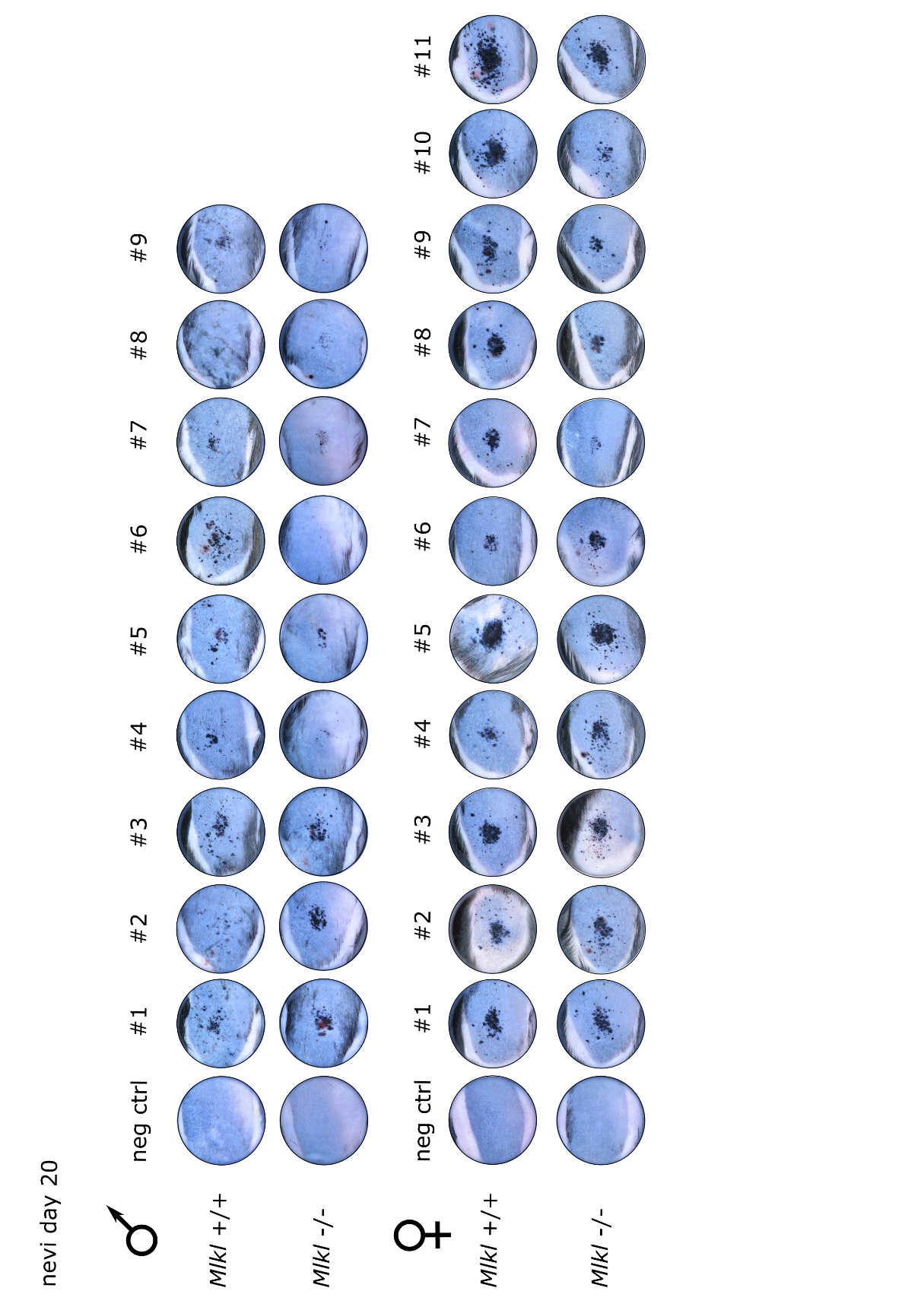


**Supplementary Figure 3. Dermatoscopic images of delayed nevi growth in *Mlkl^-/-^* male mice, which is not observed in *Mlkl^-/-^* female mice.** Dermatoscopic images of 9 (male)/11 (female) nevi per genotype group at day 20. Complete overview belonging to figure 2C. Mice with *Mlkl^+/+^* or ^-/-^ *Tyr::CreERT2^+/+^;Braf^V600Etg/+^;Pten^fl/fl^* background, challenged with 4-OHT, are indicated as negative control.


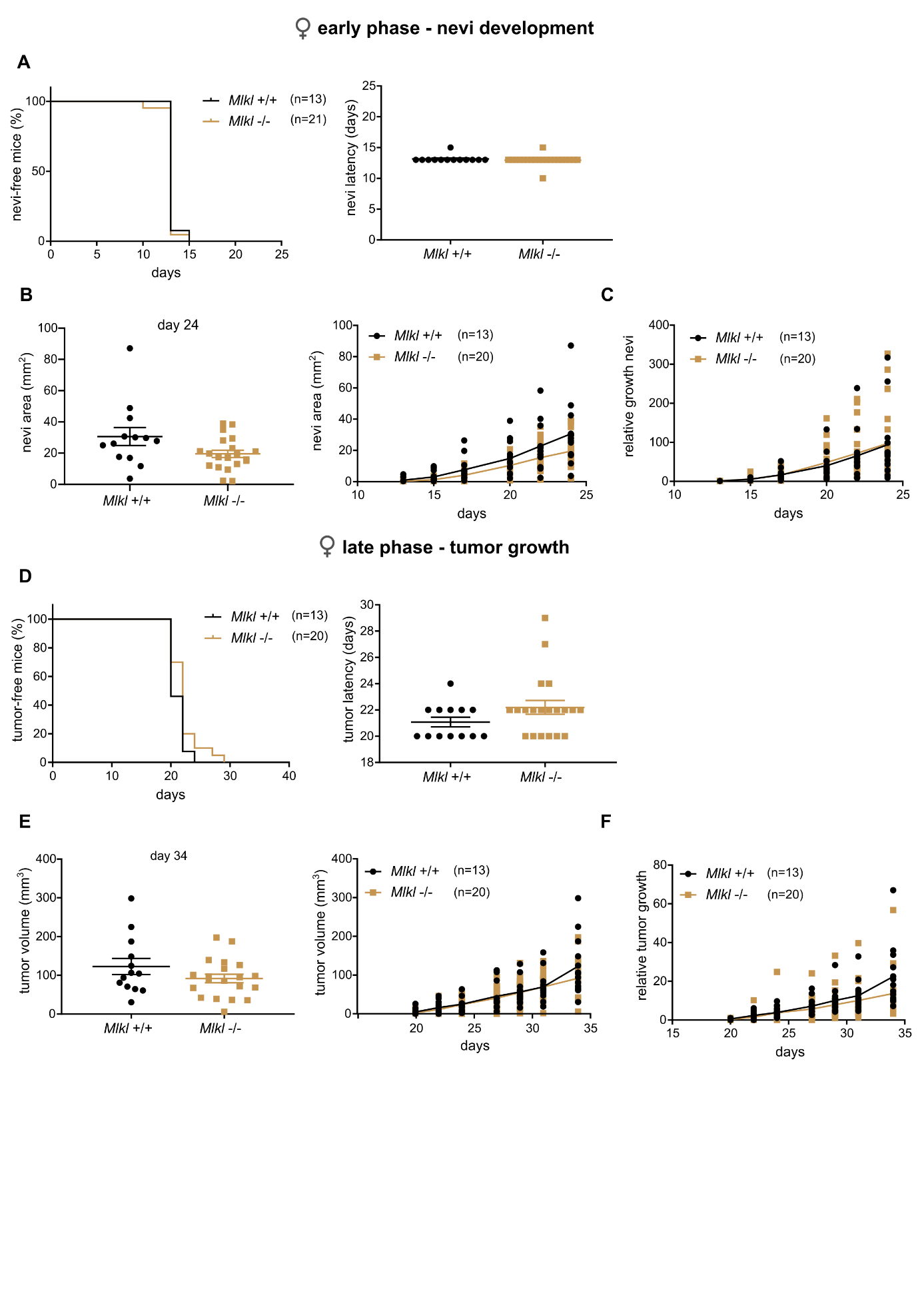


**Supplementary Figure 4. MLKL deficiency does not affect melanoma development with *Braf^V600E^Pten^-/-^* background in female mice.** Nevi (A) or tumor (D) latency and percentage of nevi/tumor-free female mice of *Mlkl^+/+^* and *Mlkl^-/-^ Braf^V600E^Pten^-/-^* background were compared. Mean value and SEM are indicated. Log-rank (mantel-cox) test and unpaired two-tailed t-test were performed respectively. Nevi area (mm^2^) (B) or tumor volume (mm^3^) were calculated using ImageJ and caliper measurements respectively. Relative nevi/tumor growth (C/F) was calculated by dividing values at every timepoint by the value of the first measurement. The line connects the mean values over time. Repeated measurement analysis was performed by maximum likelihood (REML) approach over time as described in Materials and Methods section. Nevi area (day 24) (B) and tumor volume (day 34) (E) are also illustrated (Mean value ± SEM). Unpaired two-tailed t-test was performed.


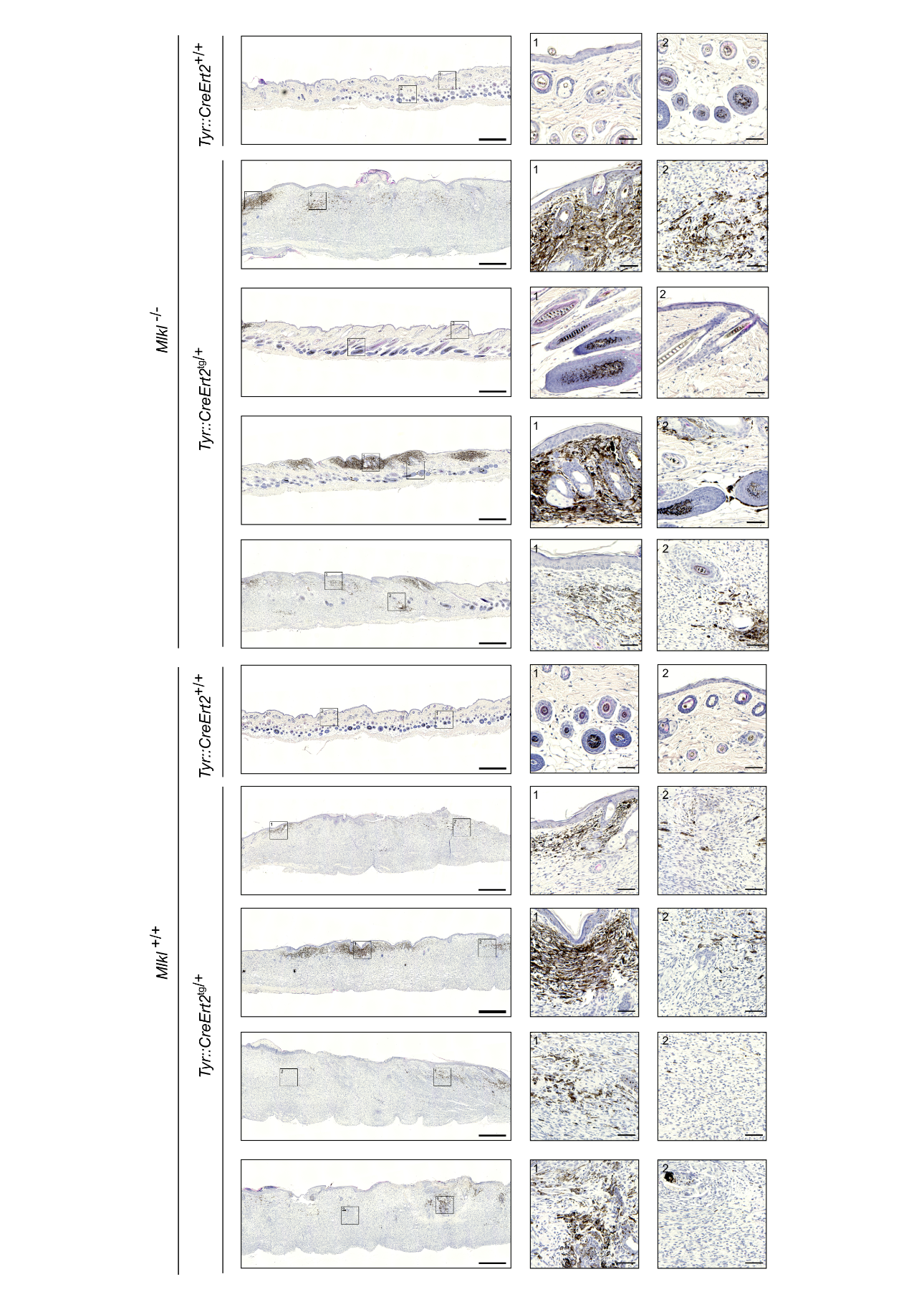


**Supplementary Figure 5. MLKL deficiency in *Braf^V600E^Pten^-/-^* male mice results in a delay in nevi development, but does not alter tumor mass architecture and intra-tumoral cell death events.** H/E staining of tumor tissue collected at day 36 after 4-OHT treatment. Extension of Figure 3. Four representative tumor sections are shown for each genotype with *Tyr::CreERT2^+/+^;Braf^V600Etg/+^;Pten^fl/fl^ or ^tg/+^* background. Images were taken with a Slide Scanner Axio Scan (Zeiss) and analyzed using ZEN (blue) software (Zeiss). Scale bar in the overview picture represents 500 µM, scale bar in the inserts represent 50 µM.


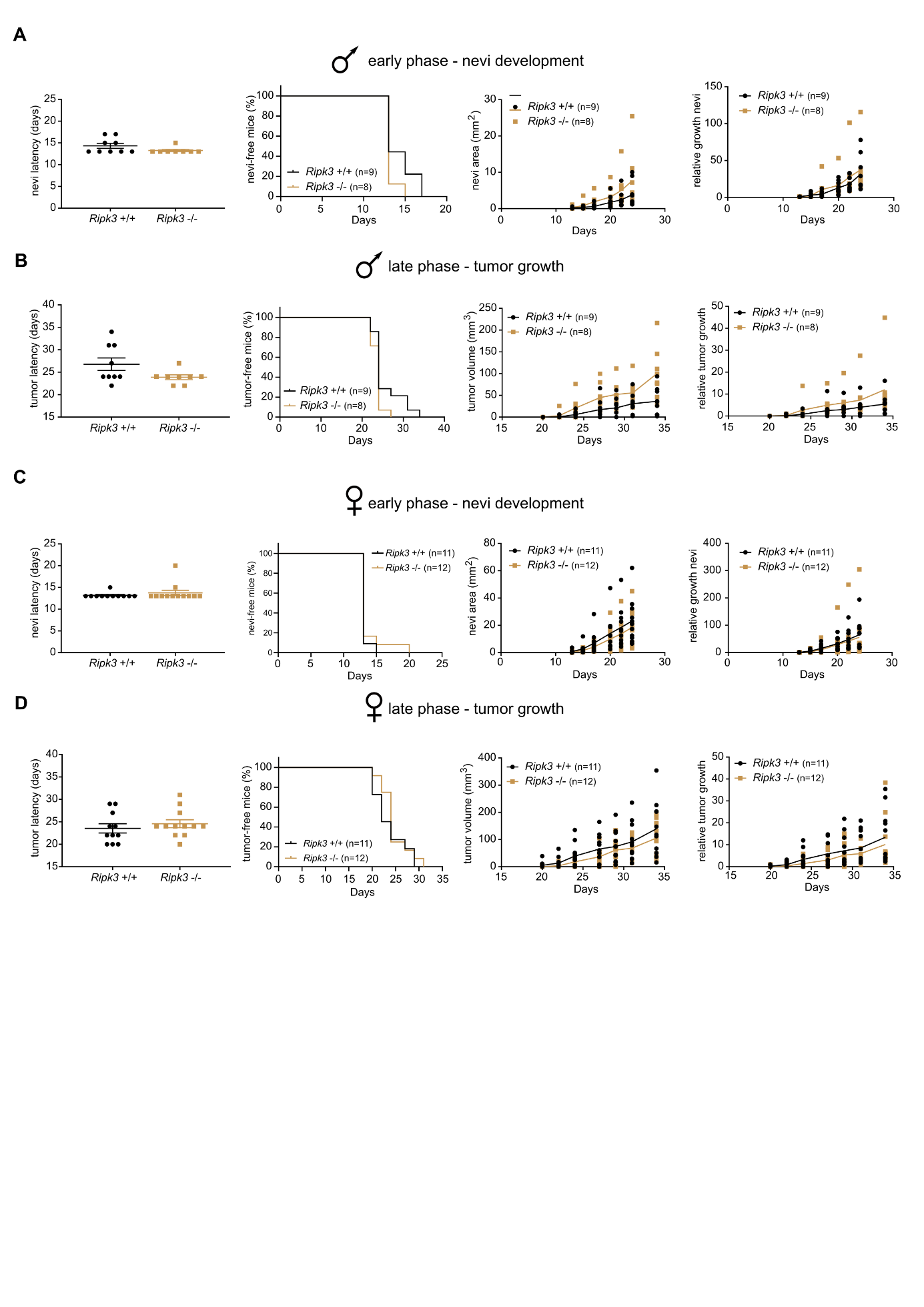


**Supplementary Figure 6. RIPK3 deficiency does not affect melanoma growth with *Braf^V600E^Pten^-/-^* background in either male or female mice.** Nevi (A,C) or tumor (B,D) latency and percentage of nevi/tumor-free mice of *Ripk3^+/+^ and Ripk3^-/-^* mice with *Braf^V600E^Pten^-/-^* background were compared, males (A-B) and females (C-D) separated. Mean value and SEM are indicated. Unpaired two-tailed t-test and log-rank (mantel-cox) test were performed respectively, but no significant result was obtained. Also nevi area (mm^2^) (A,C) or tumor volume (mm^3^) (B,D) and relative nevi (A,C) or tumor (B,D) growth were calculated using ImageJ and caliper measurements respectively. Relative nevi/tumor growth was calculated for each nevi/tumor by dividing its area/volume at every timepoint by the first measurement. The line connects the mean values over time. Repeated measurements analysis was performed as describe in the Materials and Methods section.


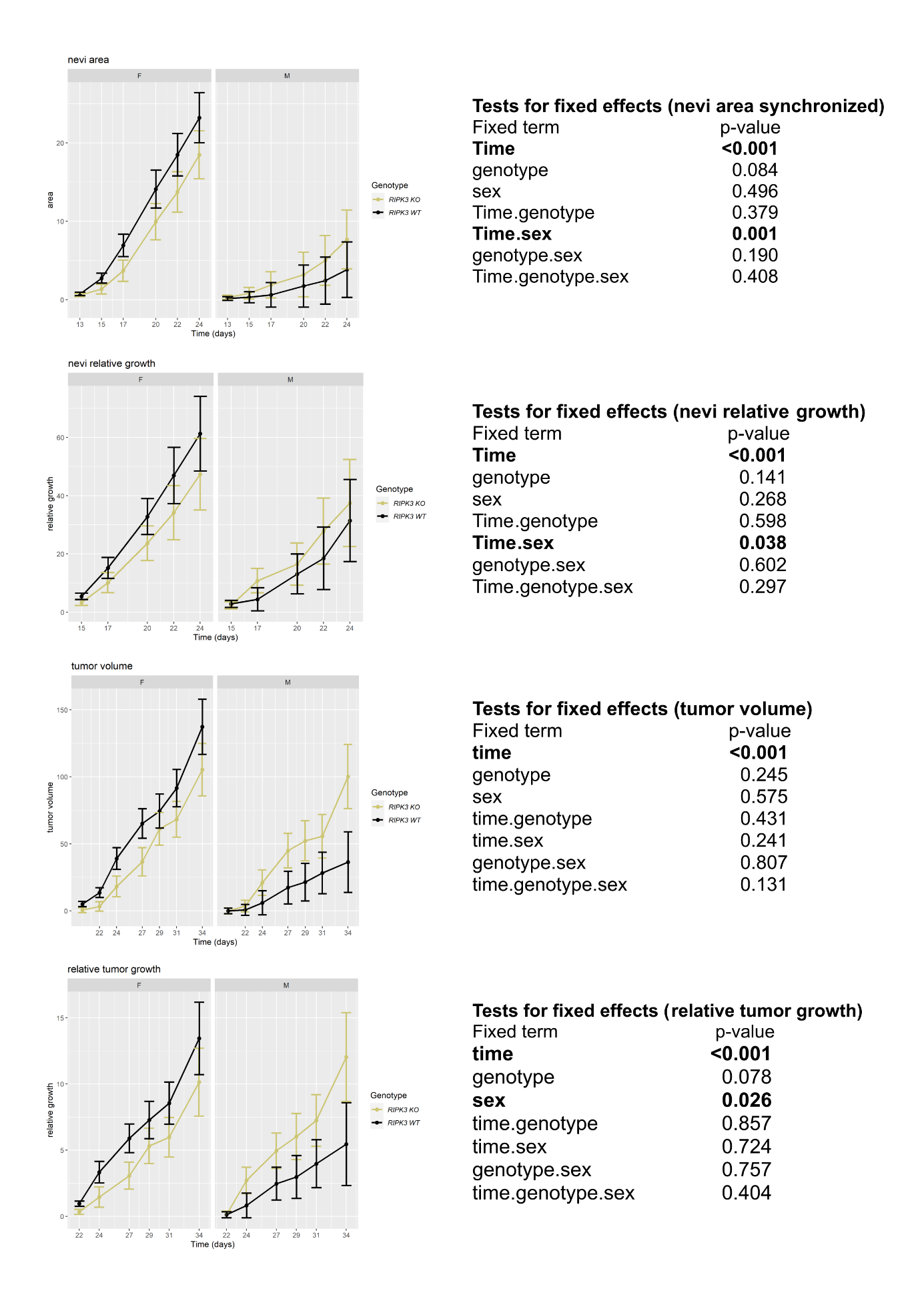


**Supplementary Figure 7. Statistical analysis performed on data from Supplementary Figure 6.** As nevi and tumor latency did not differ, data were synchronized to day 13 or day 23 respectively in order to decrease variation. Repeated measurement analysis was performed by maximum likelihood (REML) approach over time as described in Materials and Methods section. Criteria taken into account in the analysis are: cage variation, experimental variation, sex, time and genotype. P values (F pr) are indicated. Significant p-values are indicated in bold.


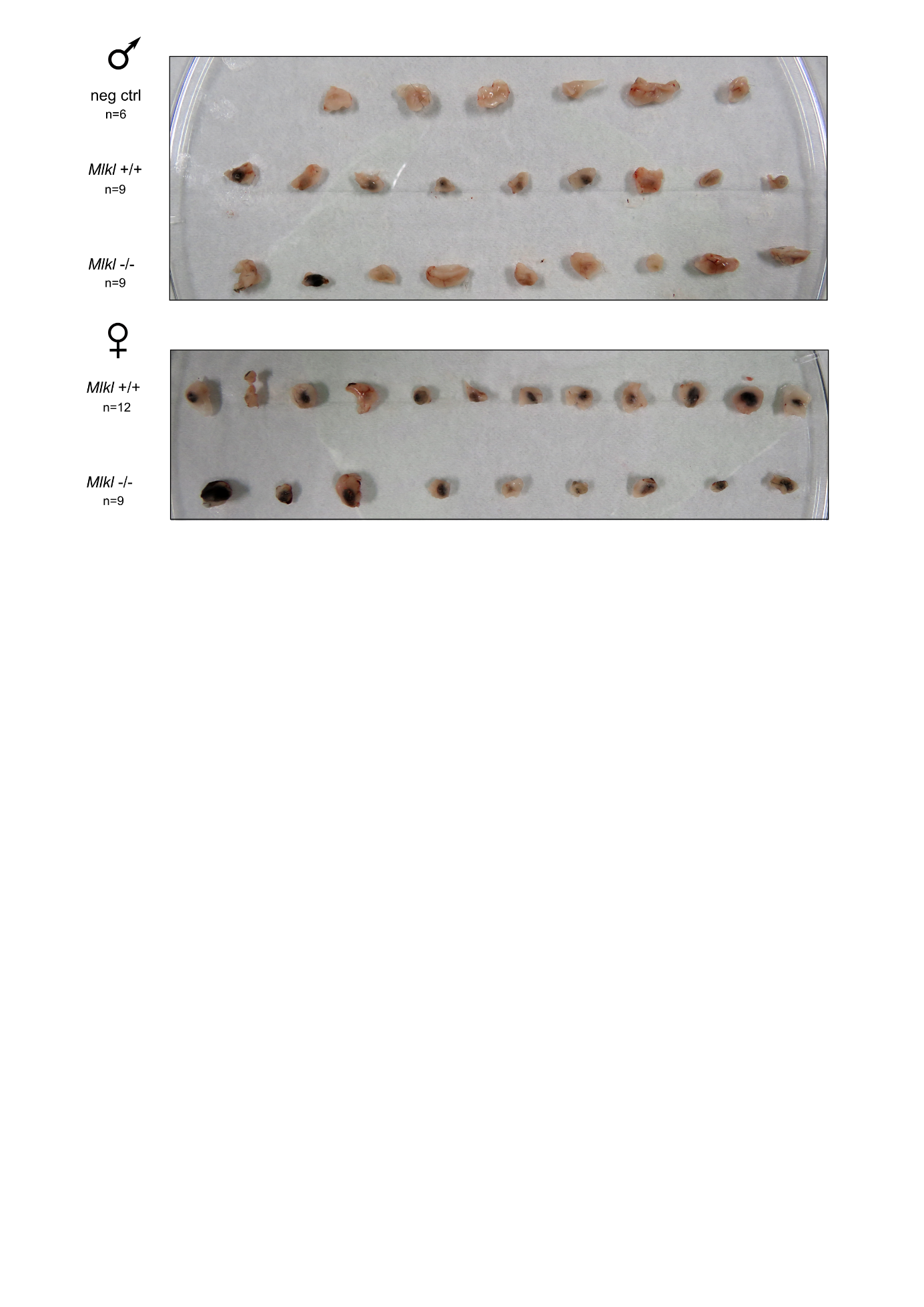


**Supplementary Figure 8. Infiltration of melanoma cells in the lymph node of male and female mice.** Macroscopic image of the lymph nodes of male and female *Mlkl^+/+^ or Mlkl^-/-^* mice, 36 days after 4-OHT treatment. Lymph node of the right flank, closest to 4-OHT-treated area, was dissected. Fat tissue surrounds the lymph node. Black color indicates the presence of differentiated melanoma cells with high melanin production. *Tyr::CreERT2^+/+^;Braf^V600Etg/+^;Pten^fl/fl^* mice treated with 4-OHT are indicated here as negative control.


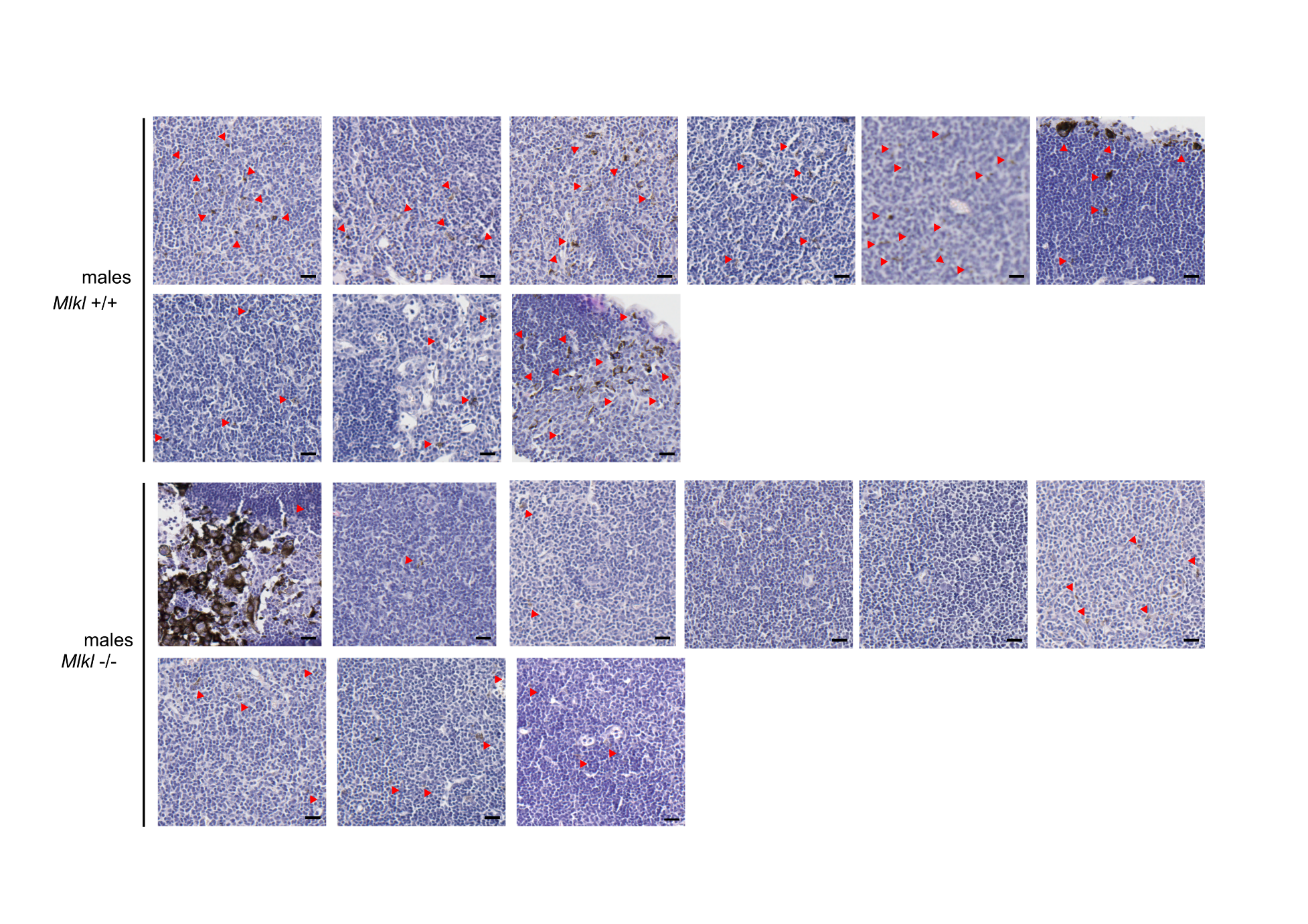


**Supplementary Figure 9. MLKL deficiency delays infiltration of melanoma cells in the lymph node in male mice with *Braf^V600E^Pten^-/-^* melanoma (histology).** Images of H/E staining of lymph node tissue collected at day 36 after 4-OHT treatment. All lymph node sections of male mice are shown, extension of Figure 4.


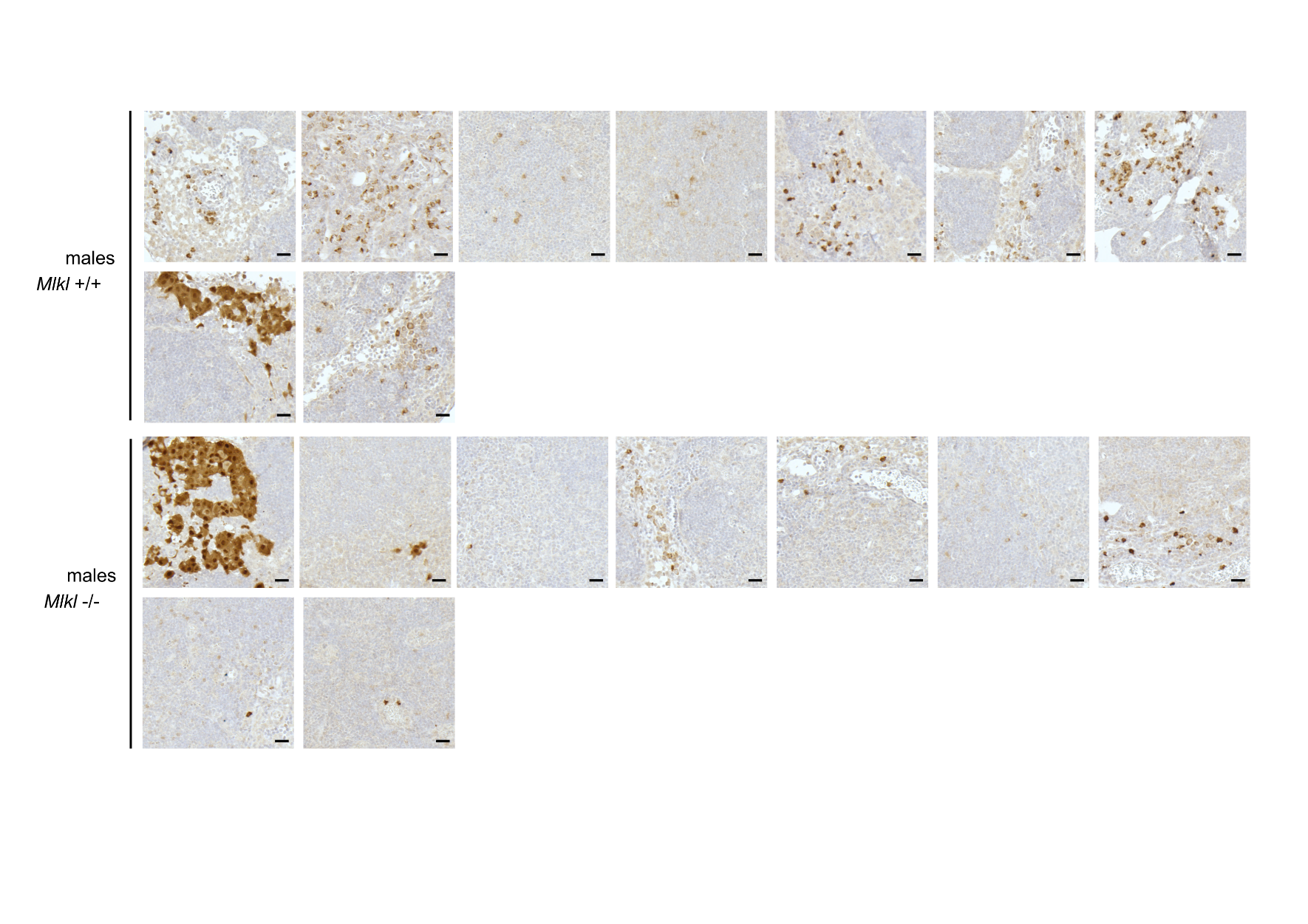


**Supplementary Figure 10. MLKL deficiency delays infiltration of melanoma cells in the lymph node in male mice with *Braf^V600E^Pten^-/-^* melanoma (immunohistochemistry).** Images of S100 staining of lymph node tissue collected at day 36 after 4-OHT treatment. All lymph node sections of male mice are shown, extension of Figure 4.


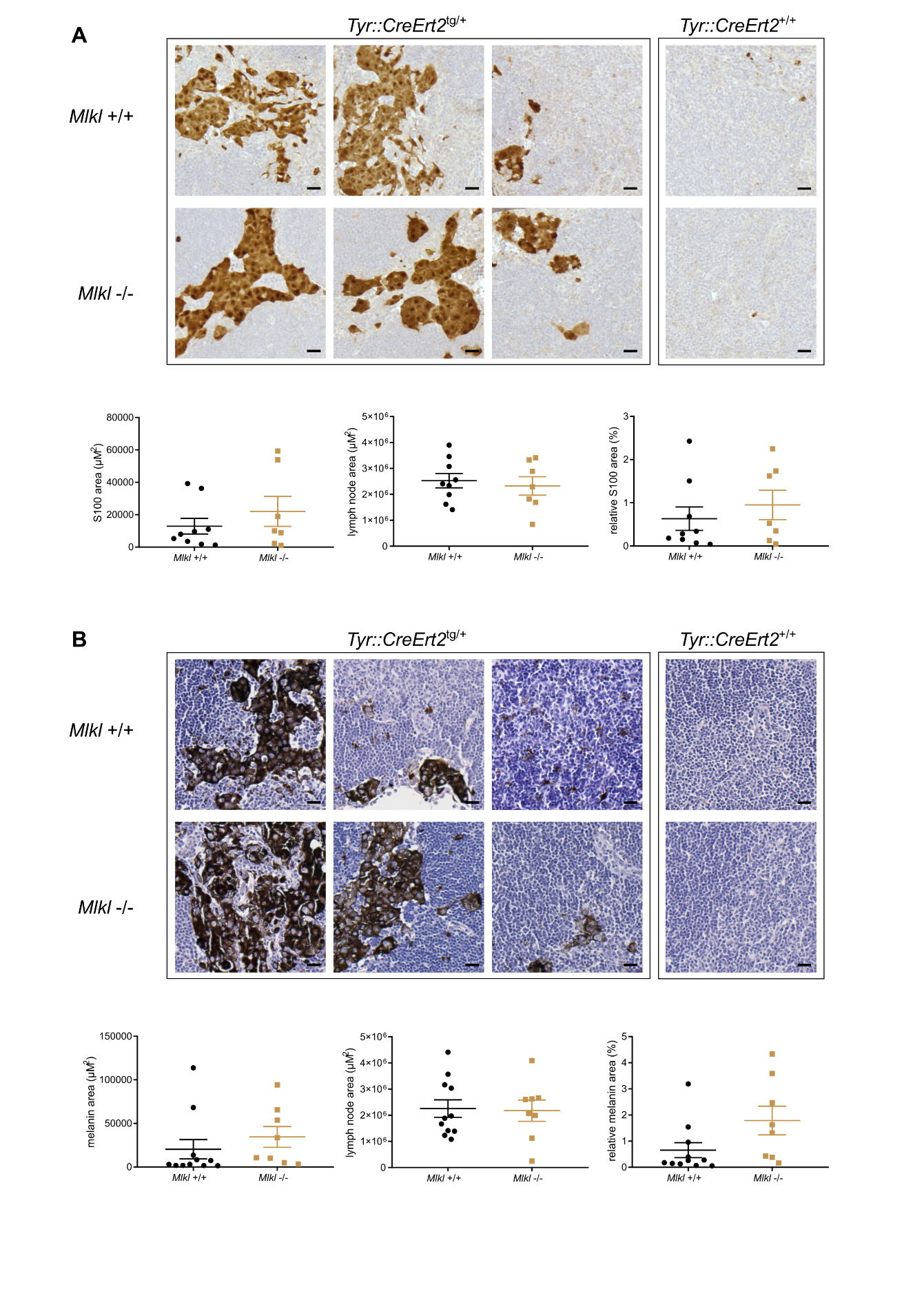


**Supplementary Figure 11. MLKL deficiency does not affect lymph node infiltration in female mice with *Braf^V600E^Pten^-/-^* melanoma.** Histological analysis and quantification of S100 staining and melanin in lymph node tissue collected at day 36 after 4-OHT treatment. Three representative lymph node sections are shown for each genotype with *Tyr::CreERT2^+/+^ or ^tg/+^;Braf^V600Etg/+^;Pten^fl/fl^* background. Images were taken with a Slide Scanner Axio Scan (Zeiss) and analyzed using ZEN (blue) software (Zeiss). Scale bar represents 20 µM. Unpaired two-tailed t-test was performed for S100/melanin area, lymph node area and relative S100/melanin area (S100/melanin area divided by lymph node area). Red arrows indicate melanin-pigmented cells.


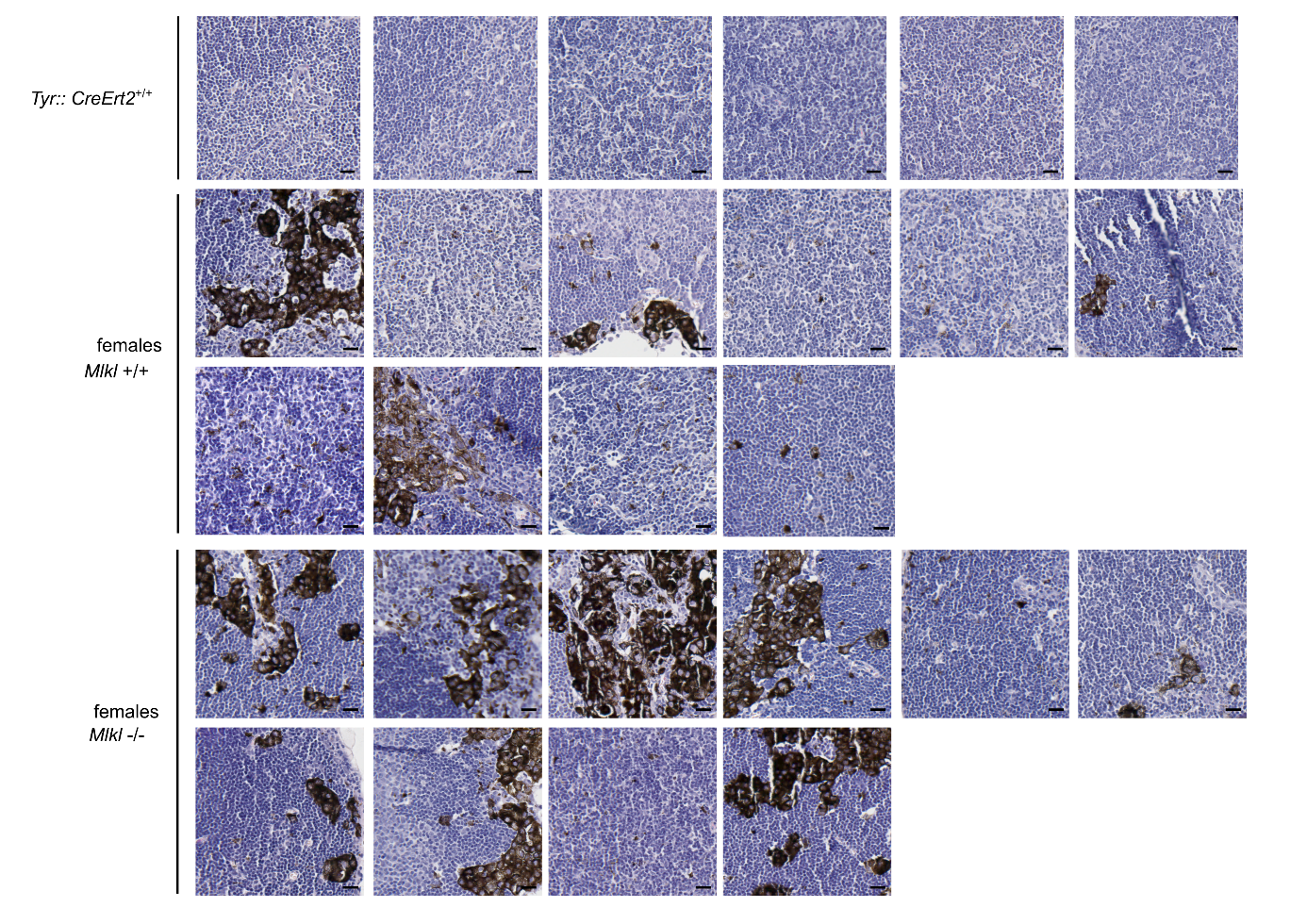


**Supplementary Figure 12. MLKL deficiency does not affect lymph node infiltration in female mice with *Braf^V600E^Pten^-/-^* melanoma (histology).** Images of H/E staining of lymph node tissue collected at day 36 after 4-OHT treatment. All lymph node sections of female mice are shown, extension of Supplementary Figure 11.


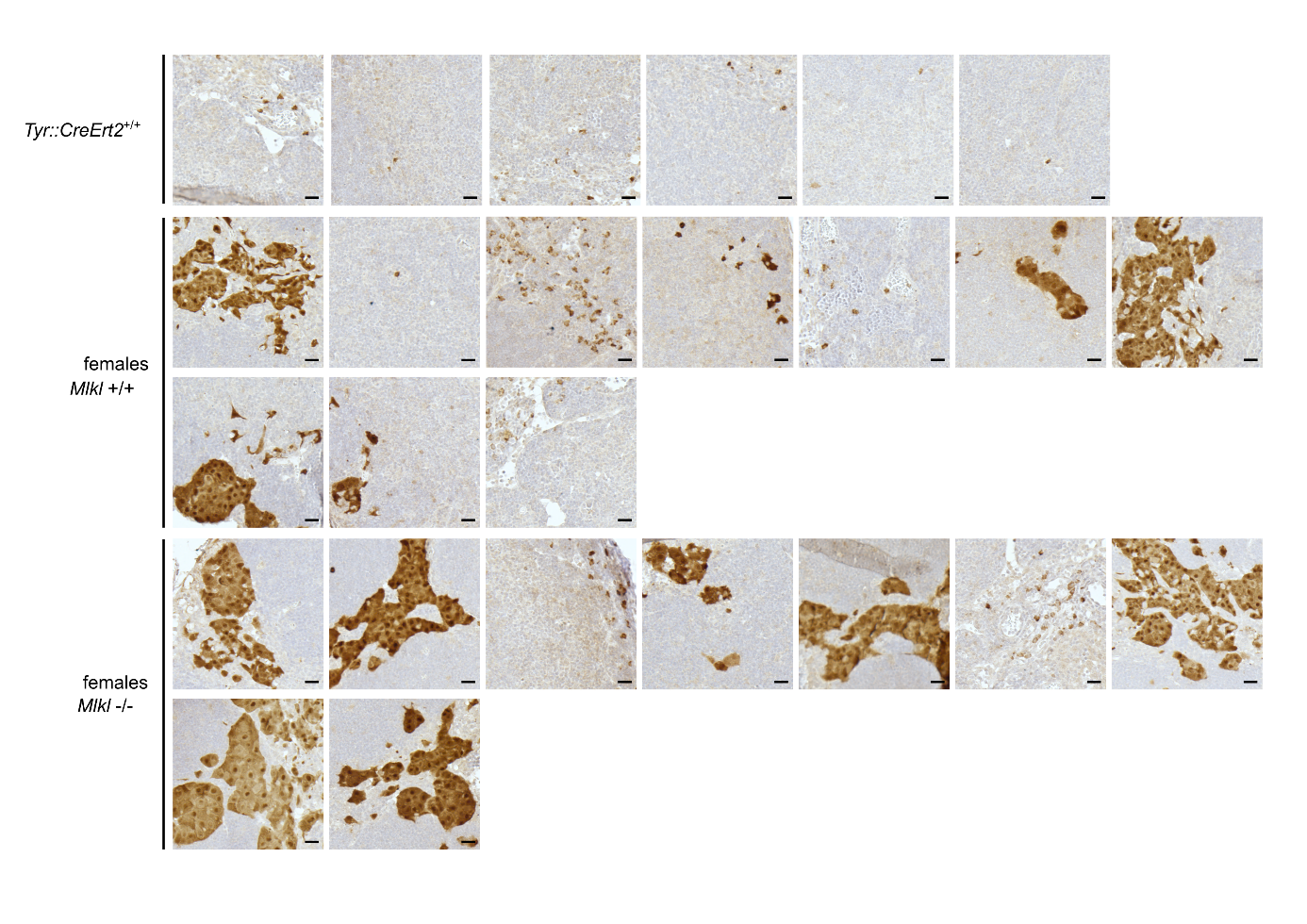


**Supplementary Figure 13. MLKL deficiency does not affect lymph node infiltration in female mice with *Braf^V600E^Pten^-/-^* melanoma (Immunohistochemistry).** Images of S100 staining of lymph node tissue collected at day 36 after 4-OHT treatment. All lymph node sections of female mice are shown, extension of Supplementary Figure 11.
